# Silica nanoparticles enhance resistance of *Vitis vinifera* to downy mildew

**DOI:** 10.1186/s12870-026-08718-0

**Published:** 2026-04-15

**Authors:** Esteban Alfonso, Augustine Jaccard, Sylvain Schnée, Amanda Malvessi Cattani, Clara Chevalley, Emilie Michellod, Michaël Farny, Robin Sonnard, Eric Remolif, Katia Gindro, Markus Rienth

**Affiliations:** 1 College of Viticulture and Enology, HES-SO Changins, University of Applied Sciences and Arts Western Switzerland, Nyon, Switzerland; 2 Agroscope Changins, Mycology Group, Nyon, Switzerland; 3 FiBL, Research Institute of Organic Agriculture, Lausanne, Switzerland

**Keywords:** Grapevine, Plant defence, Plant protection, *Plasmopara viticola*, Silica nanoparticles

## Abstract

**Background:**

Silica nanoparticles (SiO_2_ NPs) are emerging as promising tools for sustainable plant disease management. While their ability to enhance disease resistance has been demonstrated in several crop species, their potential in grapevine (*Vitis vinifera*) remains poorly investigated. Downy mildew, caused by the oomycete pathogen *Plasmopara viticola*, is one of the most destructive grapevine diseases worldwide, constituting a major challenge to viticulture. This study evaluates the efficacy of SiO_2_ NPs in controlling *P. viticola* infection and investigates the underlying plant responses at the molecular level.

**Results:**

Foliar application of SiO_2_ NPs significantly reduced *P. viticola* infection in grapevine under both controlled (leaf disc assay) and field conditions. In vitro assays showed that SiO_2_ NPs did not exert direct toxicity towards *P. viticola*, indicating that the observed protection is plant-mediated. Transcriptomic analysis of SiO_2_ NP-treated non-infected leaves revealed transient activation of defence-related genes associated with salicylic acid, jasmonic acid and ethylene signalling, secondary metabolism and transcriptional regulation. However, these transcriptional responses were markedly attenuated upon infection. Metabolite profiling of phytohormones and stilbenes showed no significant differences between SiO_2_ NP-treated and control plants, suggesting that enhanced resistance relies on other plant-derived mechanisms.

**Conclusion:**

SiO_2_ NPs effectively reduced downy mildew severity in grapevine, although the underlying transcriptional and metabolic changes responsible for this effect remain unidentified. This suggests that alternative, possibly non-transcriptional mechanisms contribute to the observed protection. Overall, these findings provide new insights into SiO_2_ NP-induced responses in grapevine and highlight their potential for sustainable disease management.

**Supplementary Information:**

The online version contains supplementary material available at 10.1186/s12870-026-08718-0.

## Background

Grapevine (*Vitis vinifera* L.) is a major crop of cultural and economic importance, but its development is consistently threatened by fungal and oomycete pathogens. *Plasmopara viticola* is the causal agent of downy mildew, which is one of the most destructive diseases affecting vineyards worldwide [1]. This obligate biotrophic oomycete infects all green tissues of grapevine, with leaves being the primary infection site, where it enters through stomata and colonises the intercellular spaces to establish infection [2]. The infection disrupts photosynthetic activity by damaging leaf tissues, which in turn limits carbohydrate availability for fruit development and can result in substantial yield losses if not properly controlled [3]. Beyond yield reduction, downy mildew can also affect fruit quality, by altering sugar accumulation, acidity and phenolic content, ultimately compromising wine quality and sensory characteristics [4].

To limit yield loss, frequent applications of synthetic fungicides throughout the growing season remain the conventional strategy, placing grapevine among the crops with the highest fungicide use per hectare [5]. The intensive use of fungicides over decades has led to widespread environmental contaminations, with residues detected in soils, water, and even agricultural products such as wine. These residues pose risks to human health and biodiversity and have contributed to the emergence of resistant pathogen populations [6]. In response, the European Commission has set an ambitious goal to reduce pesticide use by 50% before 2030 [7]. In organic viticulture, where synthetic fungicides are prohibited, downy mildew is primarily managed by copper-based formulations [8]. Although highly effective, the repeated application of copper over more than a century has led to its accumulation in vineyard soils, causing toxicity to soil microorganisms, reduced biodiversity and contamination of water sources [9, 10]. Consequently, there is an urgent need for safer and more sustainable alternatives to ensure effective disease control while minimising environmental impact [11].

In response to the environmental concerns associated with copper applications, research has focused on developing eco-friendly alternatives that can reduce or even replace its use [12]. Among these, natural compounds and inorganic substances with biostimulant or resistance-inducing properties, such as essential oils [13–15], chitosan [16], phosphonates [17], hydrolysed proteins [18] and plant extracts [19–21] have been studied for their potential to enhance grapevine resistance to *P. viticola*. These compounds have been shown to modulate grapevine defences, strengthen cell walls, or disrupt pathogen development, thereby contributing to disease reduction while minimising ecological damage.

In this context, silicon (Si) has been widely recognised for its ability to enhance plant resilience to both abiotic and biotic stresses [22]. Its protective effects against pathogens have been reported in several crops, including rice, wheat, tomato and cucumber [23]. Application of Si can reinforce plant cell walls through silica (SiO_2_) deposition in the cuticle and epidermis, forming a mechanical barrier that limits pathogen penetration [24]. Additionally, exogenous Si has been found to prime plant immune responses, leading to the enhanced production of reactive oxygen species (ROS), activation of defence genes related to the jasmonic acid (JA), ethylene (ET) and salicylic acid (SA) signalling pathways and accumulation of antimicrobial compounds [23]. Beyond its role in pathogen resistance, Si has also been shown to improve photosynthetic performance, enhance antioxidant capacity, and optimise nutrient uptake and balance [25, 26], thereby contributing to overall plant fitness and productivity.

Building on this knowledge, recent advances in nanotechnology have led to the exploration of silica nanoparticles (SiO_2_ NPs) as a promising way of Si delivery. In plants, the only bioavailable form of Si is monosilicic acid (Si(OH)_4_), which results from the hydrolytic degradation of SiO_2_ [27]. This process also underlies the mode of action of SiO_2_ NPs, which can release Si(OH)_4_ upon degradation. Due to their nanoscale size, SiO_2_ NPs can enter the leaves through stomata and accumulate in the extracellular air spaces of the spongy mesophyll and slowly release bioavailable Si to plant cells [28]. Several studies have shown that SiO_2_ NP application can trigger plant defence responses and restrict pathogen infection. In *Arabidopsis thaliana*, treatment with SiO_2_ NPs induced both local and systemic SA-dependent resistance against the bacterial pathogen *Pseudomonas syringae* [28]. Similarly, Wang et al. (2022) [29] reported that treatment with SiO_2_ NPs reduced bacterial wilt in tomato by scavenging ROS and activating SA-related defence genes. In rice, foliar treatment with SiO_2_ NPs has been shown to stimulate immunity and enhance resistance against rice blast in an SA-dependent manner [30].

In contrast, the potential of SiO_2_ NPs to manage grapevine diseases remains poorly investigated. One recent study reported that field applications of SiO_2_ NPs on *V. vinifera* cv. Thompson Seedless reduced downy mildew severity, an effect associated with the induction of several defence-related genes from the JA, ET and SA pathways, and with stomatal closure [31]. In this study, we aimed to further characterise the effects of SiO_2_ NP application on downy mildew development in grapevine, focusing on transcriptomic and metabolomic profiling to uncover SiO_2_ NP-induced molecular responses in both non-infected and *P. viticola*-infected tissues. To this end, we combined leaf disc assays and multi-year field trials to evaluate the impact of foliar SiO_2_ NP treatments, and we additionally assessed the potential direct toxicity of SiO_2_ NPs towards the pathogen.

## Results

### Treatment with SiO_2_ NPs reduces *P. viticola* infection

**Fig. 1:**
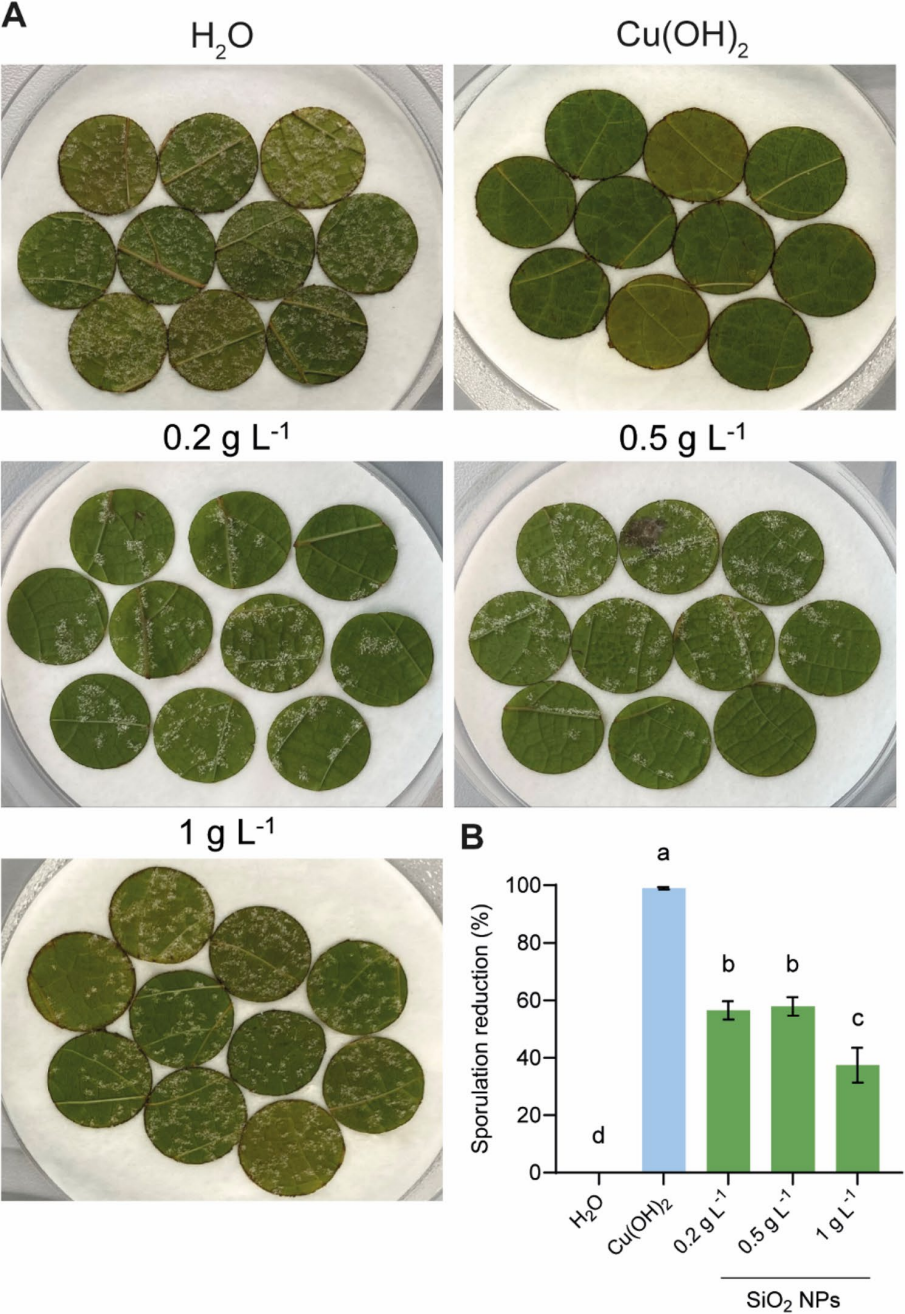
Preventive effects of SiO_2_ NPs on *P. viticola* infection in leaf discs. **A** Representative images of sporulating leaf discs treated with water (H_2_O), copper hydroxide (Cu(OH)_2_) or SiO_2_ NPs at different concentrations. **B** Quantification of sporulating area at 7 dpi following preventive application of SiO_2_ NPs. Data represent means ± SE of three independent experiments (*n* = 20 per experiment). Different letters indicate significant differences at *P* < 0.05 (One-Way ANOVA followed by Tukey’s Honest Significant Difference post hoc test).

To evaluate whether SiO_2_ NPs can induce resistance to *P. viticola* in *V. vinifera* cv. Cabernet Sauvignon, leaf discs were treated with water, copper hydroxide (Cu(OH)_2_) and increasing concentrations of SiO_2_ NPs. Twenty-four hours after application, leaf discs were infected with *P. viticola* and sporulating areas were quantified 7 days post-infection (dpi) to determine the percentage reduction of sporulation (Fig. 1A). Treatments with SiO_2_ NPs at 0.2 g L^− 1^ and 0.5 g L^− 1^ resulted in significant sporulation reductions of 56.5% and 57.9% compared to the water-treated control, respectively (Fig. 1B). Interestingly, application of SiO_2_ NPs at 1 g L^− 1^ was significantly less effective than lower concentrations, with a reduced sporulation of 37.4%. As expected, treatment with Cu(OH)_2_ completely inhibited *P. viticola* development. Based on these results, the concentration of 0.2 g L^− 1^ was selected for subsequent experiments as it significantly reduced sporulation while requiring minimal input.

### Field efficacy of SiO_2_ NPs

**Fig. 2:**
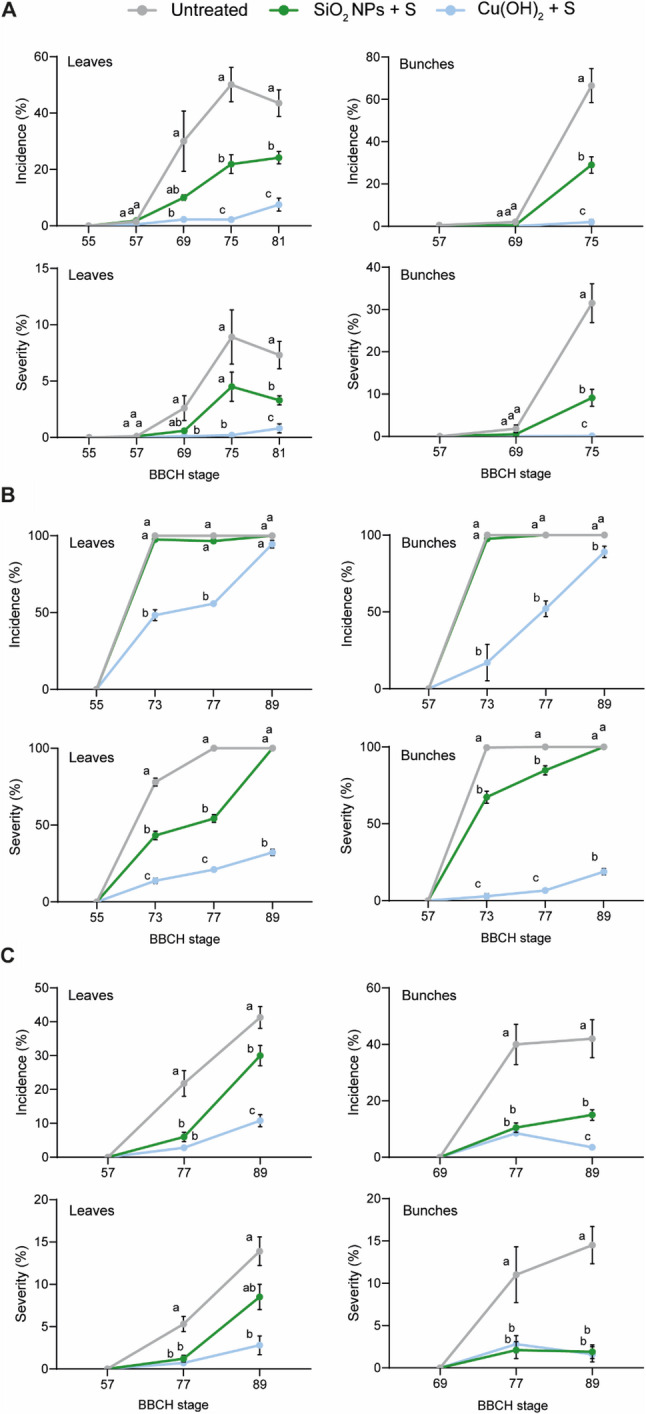
Disease incidence and severity of downy mildew in *V. vinifera* cv. Chasselas from field experimental trials in (**A**) 2023, (**B**) 2024 and (**C**) 2025. For each BBCH stage (sampling date), data represent means ± SE of four biological replicates (blocks). Each block assessment consisted of 100 leaves and 50 bunches. Different letters indicate significant differences at *P* < 0.05 (One-Way ANOVA followed by Tukey’s Honest Significant Difference post hoc test)

The efficacy of SiO_2_ NP treatments under vineyard conditions was monitored over three growing seasons (2023–2025). In 2023, despite a wet spring and conditions favourable to primary infection, overall disease pressure remained low throughout the season. During the season, disease development was recorded at standard phenological stages (BBCH; see Methods). At BBCH 57 (1 June), the first symptoms of downy mildew appeared on leaves in the untreated control plots (Fig. 2A). Although disease incidence increased progressively with vine development up to BBCH 75 (11 July), severity in the leaf canopy remained below 10%. On bunches, symptoms appeared from BBCH 69 (19 June) and reached moderate levels at the final assessment (BBCH 75), with a mean severity of 31.5% in the untreated control, sufficient to discriminate between treatments (Fig. 2A). Further evaluations were not possible because the trial was affected by a hail event that damaged growing bunches, preventing reliable assessments of downy mildew symptoms. Application of SiO_2_ NPs at 0.2 g L^− 1^ supplemented with sulfur (to ensure powdery mildew control) reduced the incidence and severity of downy mildew on leaves by approximately half compared with the untreated control (55% and 54% efficacy, respectively), while the organic reference (Cu(OH)_2_ + sulfur) achieved 91% and 95% efficacy (Table 1). On bunches, the overall efficacy of SiO_2_ NPs in reducing incidence and severity reached 57% and 71%, respectively (Table 1). These efficacy values correspond to reductions in the area under the disease progress curve (AUDPC), which integrates disease intensity over time to reflect cumulative disease development [32].

**Table 1:** AUDPC values and seasonal efficacy of SiO_2_ NPs against *P. viticola* in field trials (2023–2025)

**2023**					
**Incidence on leaves**	AUDPC^1^	Efficacy (%)	**Severity on leaves**	AUDPC^1^	Efficacy (%)
Untreated	2810 **a**	-	Untreated	435 **a**	-
SiO_2_ NPs + S	1271 **b**	55	SiO_2_ NPs + S	198 **b**	54
Cu(OH)_2_ + S	246 **c**	91	Cu(OH)_2_ + S	22 **c**	95
**Incidence on bunches**	AUDPC^1^	Efficacy (%)	**Severity on bunches**	AUDPC^1^	Efficacy (%)
Untreated	776 **a**	-	Untreated	381 **a**	-
SiO_2_ NPs + S	334 **b**	57	SiO_2_ NPs + S	110 **b**	71
Cu(OH)_2_ + S	22 **c**	97	Cu(OH)_2_ + S	1 **c**	100
**2024**					
**Incidence on leaves**	AUDPC^1^	Efficacy (%)	**Severity on leaves**	AUDPC^1^	Efficacy (%)
Untreated	8500 **a**	-	Untreated	7916 **a**	-
SiO_2_ NPs + S	8311 **a**	2	SiO_2_ NPs + S	5390 **b**	32
Cu(OH)_2_ + S	5451 **b**	36	Cu(OH)_2_ + S	1855 **c**	77
**Incidence on bunches**	AUDPC^1^	Efficacy (%)	**Severity on bunches**	AUDPC^1^	Efficacy (%)
Untreated	8150 **a**	-	Untreated	8139 **a**	-
SiO_2_ NPs + S	8093 **a**	1	SiO_2_ NPs + S	6863 **b**	16
Cu(OH)_2_ + S	4303 **b**	47	Cu(OH)_2_ + S	739 **c**	91
**2025**					
**Incidence on leaves**	AUDPC^1^	Efficacy (%)	**Severity on leaves**	AUDPC^1^	Efficacy (%)
Untreated	2176 **a**	-	Untreated	636 **a**	-
SiO_2_ NPs + S	1131 **b**	48	SiO_2_ NPs + S	298 **ab**	53
Cu(OH)_2_ + S	433 **c**	80	Cu(OH)_2_ + S	110 **b**	83
**Incidence on bunches**	AUDPC^1^	Efficacy (%)	**Severity on bunches**	AUDPC^1^	Efficacy (%)
Untreated	2797 **a**	-	Untreated	854 **a**	-
SiO_2_ NPs + S	848 **b**	70	SiO_2_ NPs + S	138 **b**	84
Cu(OH)_2_ + S	440 **b**	84	Cu(OH)_2_ + S	155 **b**	82

Different letters in bold indicate statistical difference at *P* < 0.05 (One-Way ANOVA followed by Tukey’s honest significant difference post hoc test)

^1^ Area under the disease progress curve

The 2024 season was particularly favourable for downy mildew development, with 59 days of rainfall during the trial period (cumulative 314 mm) and 47 infection events recorded (Agrometeo, www.agrometeo.ch). The first symptoms appeared on the foliage at BBCH 55 (2 June) in the untreated control and progressed rapidly, reaching full canopy infection at BBCH 73 (2 July) with a severity of 78% (Fig. 2B). Application of SiO_2_ NPs reduced disease severity on leaves by approximately half at BBCH 73 and 77 (assessments on 2 and 25 July), but disease continued to develop after the last treatment on 6 August, leading to complete foliar destruction by BBCH 89 (10 September). In contrast, the organic reference could maintain disease severity between 13% and 32% during the season (Fig. 2B). On bunches, the first symptoms appeared at BBCH 57 (9 June) in the untreated control, with severity reaching 100% at BBCH 73, highlighting strong epidemic dynamics driven by regular rainfall, favourable temperature and multiple infection events during the season. SiO_2_ NP application significantly reduced disease severity at BBCH 73 and 77, corresponding to overall AUDPC-based efficacies of 32% on leaves and 16% on bunches, although it did not prevent total bunch destruction by the end of the trial (Fig. 2B). The organic reference achieved a final efficacy of 77% on leaves and 91% on bunches (Table 1).

The 2025 season was characterised by regular rainfall in spring, favourable to primary infections, followed by a prolonged dry period in June, which limited the disease spread. The first symptoms on leaves appeared at BBCH 57 (4 June) and remained low, with a mean severity of approximately 14% on both leaves and bunches at the last assessment (BBCH 89, 4 September) (Fig. 2C). Several infection events occurred after the final treatment of SiO_2_ NPs in mid-July, which may explain the moderate protection observed on foliage (53% efficacy). However, the efficacy on bunches (84%) was identical to that of the organic reference (Table 1).

### SiO_2_ NPs do not exert direct toxicity against *P. viticola*

**Fig. 3:**
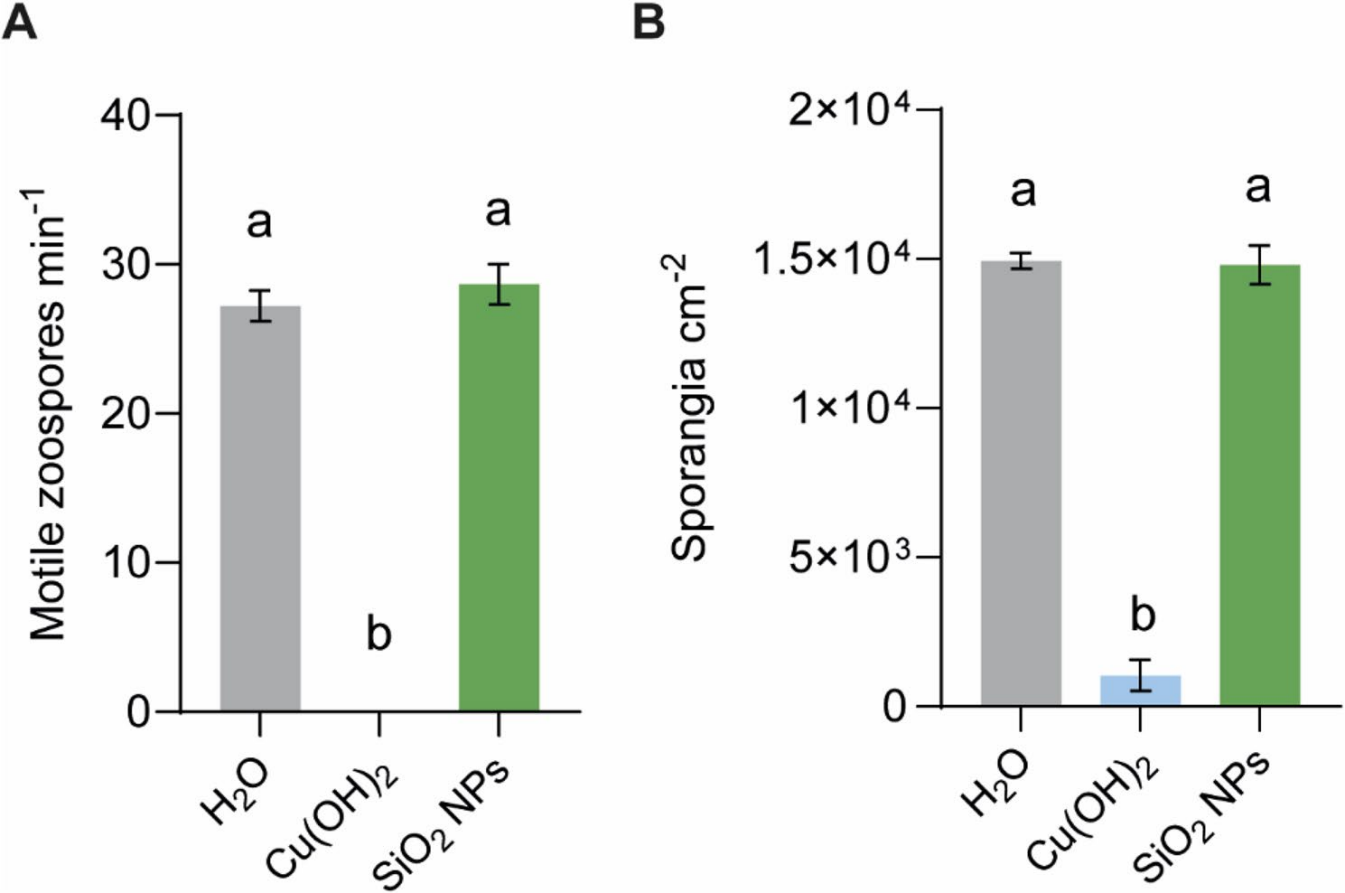
Effects of SiO_2_ NPs on *P. viticola* zoospores viability and infectivity. **A** Motile zoospores per min counted after incubation of sporangia with the different treatments. Data represent means ± SE of three independent experiments (*n* = 9 per experiment). Different letters indicate significant differences at *P* < 0.05 (One-Way ANOVA followed by Tukey’s Honest Significant Difference post hoc test). **B** Number of sporangia per cm^2^ of leaf tissue retrieved from sporulating leaf discs. Data represent means ± SE of three independent experiments (*n* = 20 per experiment). Different letters indicate significant differences at *P* < 0.05 (One-Way ANOVA followed by Tukey’s Honest Significant Difference post hoc test)

To determine whether the reduction in *P. viticola* infection following SiO_2_ NP treatment results from a direct toxic effect on the pathogen, the impact of SiO_2_ NPs on sporangial viability was assessed. Sporangial germination was evaluated by monitoring the release of motile zoospores after incubation for 2 h with SiO_2_ NPs. Zoospore release was comparable to the water-treated control, indicating that SiO_2_ NPs did not impair sporangial germination. In contrast, incubation with Cu(OH)_2_ completely inhibited zoospore motility (Fig. 3A). We next investigated whether SiO_2_ NP exposure affected pathogen infectivity. Sporangia previously incubated with SiO_2_ NPs were used to inoculate healthy leaf discs, and disease development was quantified at 7 dpi by measuring sporangia production. Leaf discs inoculated with SiO_2_ NP-treated sporangia exhibited infection levels similar to those observed in the water-treated control, whereas sporangia incubated with Cu(OH)_2_ failed to initiate infection (Fig. 3B). Collectively, these results demonstrate that SiO_2_ NPs do not exert direct toxicity against *P. viticola* under the tested conditions.

### Subcellular distribution of SiO_2_ NPs within *V. vinifera* leaves

**Fig. 4:**
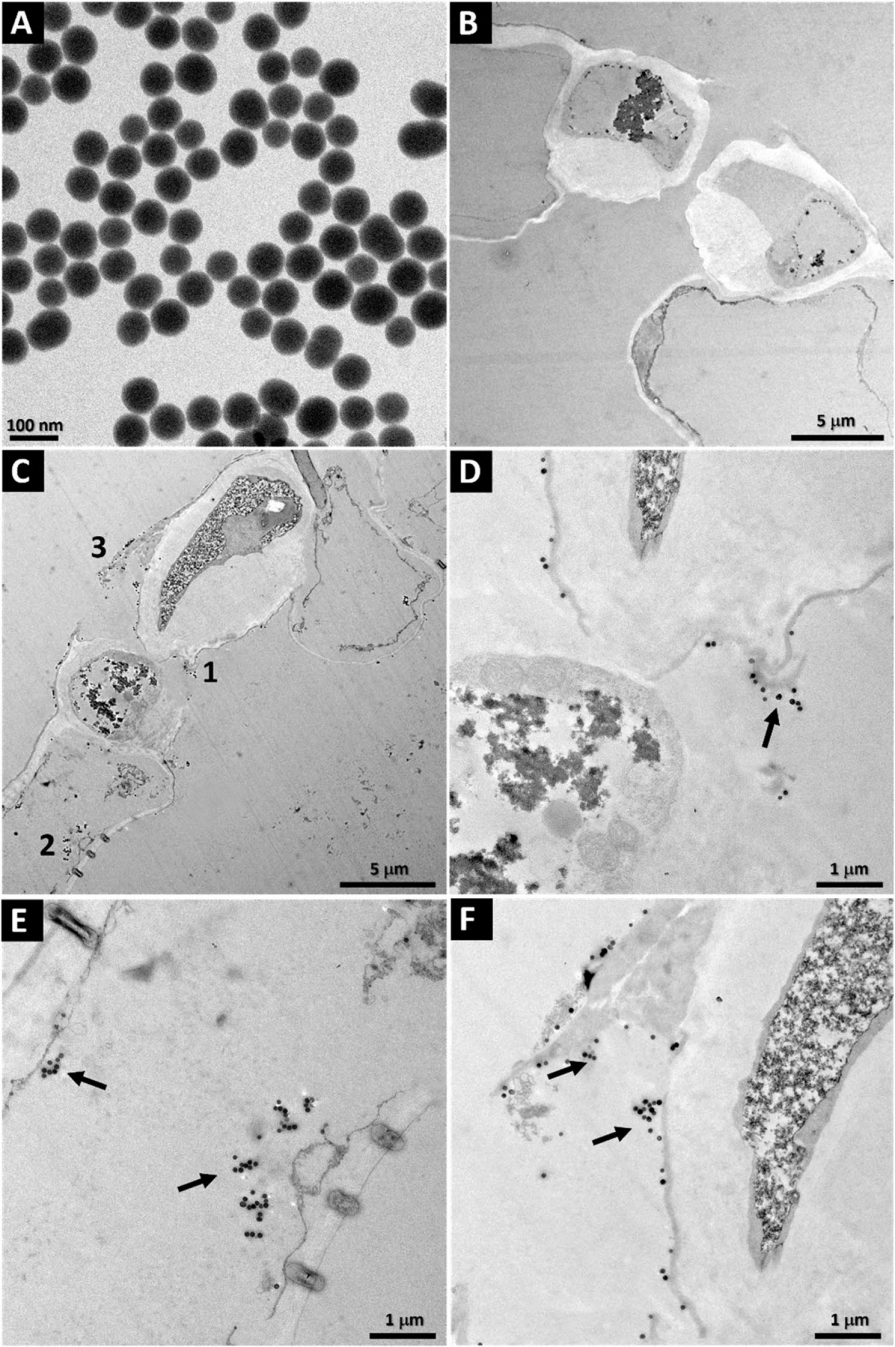
Subcellular localisation of SiO_2_ NPs in grapevine leaves. Leaves were sprayed with water (control) or SiO_2_ NPs and examined by TEM 24 h after application. **A** SiO_2_ NPs used in this study. **B** Stomata of a water-treated control leaf. **C** Stomata of a SiO_2_ NP-treated leaf with visible electron-dense particles. Numbered regions in (**C**) indicate magnified views of SiO_2_ NPs in (**D**) the substomatal chamber (1), (**E**) an adjacent epidermal cell (2) and (**F**) the cuticular surface at the stomatal aperture (3). Black arrows indicate SiO_2_ NPs

To better understand how SiO_2_ NPs were distributed within the cells of grapevine leaves, transmission electron microscopy (TEM) was used for particle visualisation. The SiO_2_ NPs in solution were well dispersed (Fig. 4A), with an average particle size of 54 ± 7 nm, as previously reported [28]. Detached leaves were sprayed with SiO_2_ NPs and examined 24 h after application. In control leaves sprayed with water, epidermal cells and stomata displayed normal ultrastructure, and substomatal chamber contained no electron-dense structures (Fig. 4B). In contrast, leaves treated with SiO_2_ NPs showed electron-dense spherical particles corresponding to SiO_2_ NPs (Fig. 4C). Particles were detected at multiple sites: within the substomatal chamber (Fig. 4D), inside the epidermal cell adjacent to the stomata (Fig. 4E) and at the cuticular surface near the stomatal aperture (Fig. 4F). These observations confirm that SiO_2_ NPs can access internal leaf spaces following foliar application, primarily through stomatal openings.

### Global transcriptomic shifts induced by SiO_2_ NPs and *P. viticola* infection

**Fig. 5:**
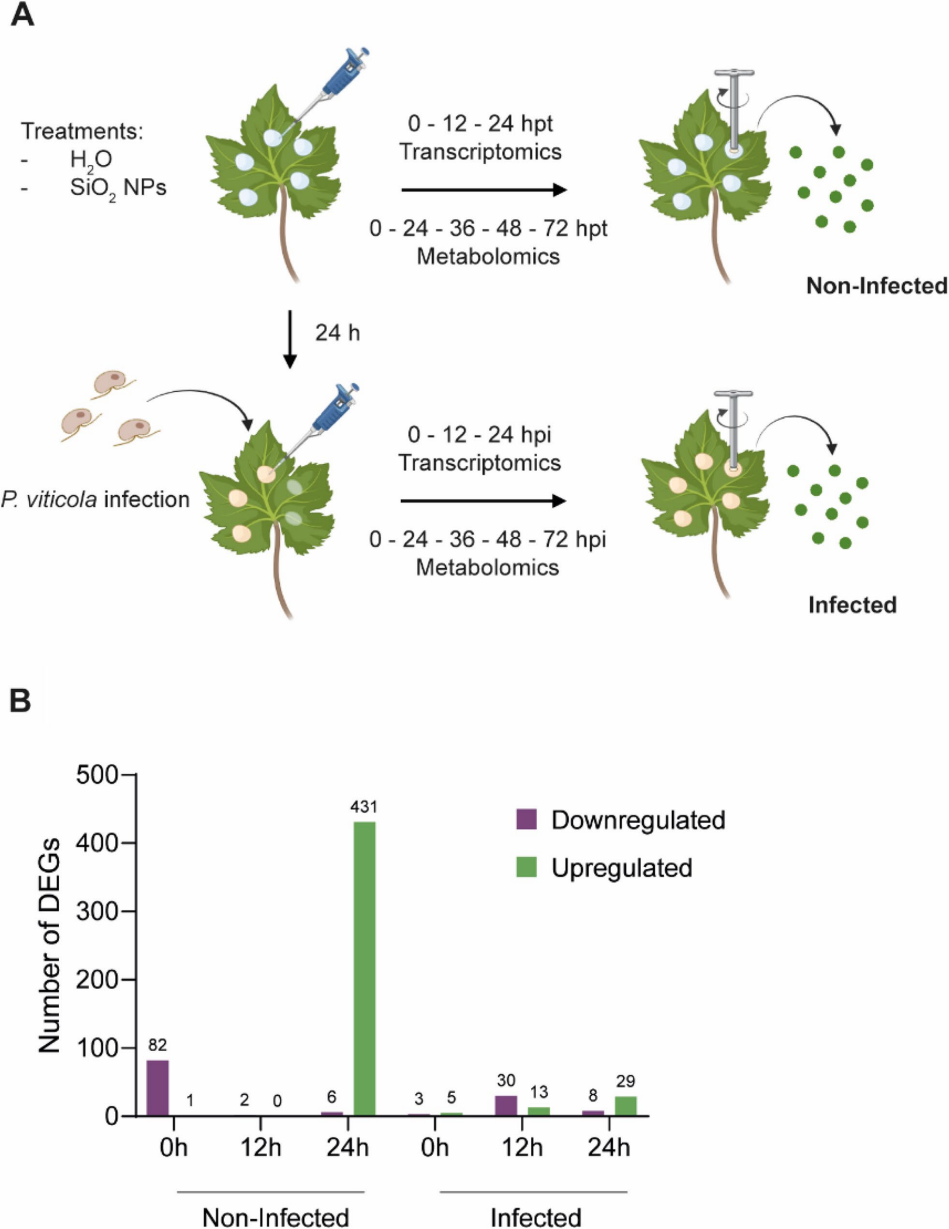
Experimental design and overview of differentially expressed genes. **A** Schematic representation of the experimental setup for transcriptomic and metabolomic analyses. Leaves were treated with either water (H_2_O) or SiO_2_ NPs and samples were harvested at 0, 12 and 24 h post-treatment (hpt) for transcriptomic analysis and at 0, 24, 36, 48 and 72 hpt for metabolomic analysis (non-infected samples). In parallel, leaves were pre-treated with either water (H_2_O) or SiO_2_ NPs for 24 h and subsequently infected with *P. viticola*. Samples were harvested at 0, 12 and 24 h post-infection (hpi) for transcriptomic analysis and at 0, 24, 36, 48 and 72 hpi for metabolomic analysis (infected samples). **B** Number of DEGs identified in each condition and time point. DEGs were defined as genes with |log_2_(FC)| > 1 and *P* < 0.05, comparing SiO_2_ NP-treated samples to water-treated controls under non-infected and infected conditions

Given that SiO_2_ NPs did not display direct toxicity towards *P. viticola* and can access internal leaf spaces through stomatal openings, we hypothesised that their protective effect may involve the activation of host immune responses. To gain insight into the underlying molecular mechanisms, we conducted a transcriptomic analysis (RNA-seq) on grapevine leaves treated with SiO_2_ NPs, with and without subsequent infection by *P. viticola*. Detached leaves were treated with either water or SiO_2_ NPs, and leaf discs were collected at 0, 12, and 24 h post-treatment (hpt) to assess treatment effects alone (non-infected). In parallel, leaves were pre-treated, inoculated 24 hpt with *P. viticola* and leaf discs were collected at 0, 12, and 24 h post-infection (hpi) to assess treatment effects on infection development (infected). The experimental design is illustrated in Fig. 5A.

To explore the global transcriptional response of grapevine leaves, a principal component analysis (PCA) was performed on the expression profiles of 20,080 filtered genes across all conditions. The two principal components, PC1 and PC2, accounted for 20.86% and 15.61% of the total variance, respectively (Supplementary Fig. 1A). The PCA revealed a clear separation between infected and non-infected samples along PC1, indicating that *P. viticola* infection is the primary factor driving transcriptional variation. Within each infection status, replicates clustered tightly, supporting the consistency of the RNA-seq data. However, the PCA analysis did not reveal clear clustering between treated and control samples across all time points, suggesting that time and infection status have a greater impact on gene expression profiles than SiO_2_ NP treatment.

Gene expression levels were analysed by comparing read counts from SiO_2_ NP-treated leaves to water-treated controls at each time point and under both infected and non-infected conditions. Across all conditions and time points, a total of 505 unique differentially expressed genes (DEGs) were identified (Supplementary Table 1). In non-infected samples, SiO_2_ NPs induced a strong transcriptional response at 24 hpt with 437 DEGs, of which 6 were downregulated and 431 upregulated (Fig. 5B). In contrast, fewer DEGs were observed at 0 and 12 hpt, with 83 genes (82 downregulated and 1 upregulated) and 2 downregulated genes, respectively. Notably, upregulated and downregulated genes were uniquely regulated at their respective time points, with no overlap between time points, as shown in the Venn diagrams (Supplementary Fig. 1B). However, from the 82 genes found downregulated immediately (0 hpt) following SiO_2_ NPs application, 76 were upregulated at 24 hpt (Supplementary Table 1). In infected samples, 8 DEGs (3 downregulated and 5 upregulated), 43 DEGs (30 downregulated and 13 upregulated) and 37 DEGs (8 downregulated and 29 upregulated) were identified at 0, 12 and 24 hpi, respectively (Fig. 5B). The overlap between time points was minimal, with only 4 genes commonly upregulated at both 12 and 24 hpi (Supplementary Fig. 1B).

### SiO_2_ NPs trigger defence-associated transcriptional responses

**Fig. 6:**
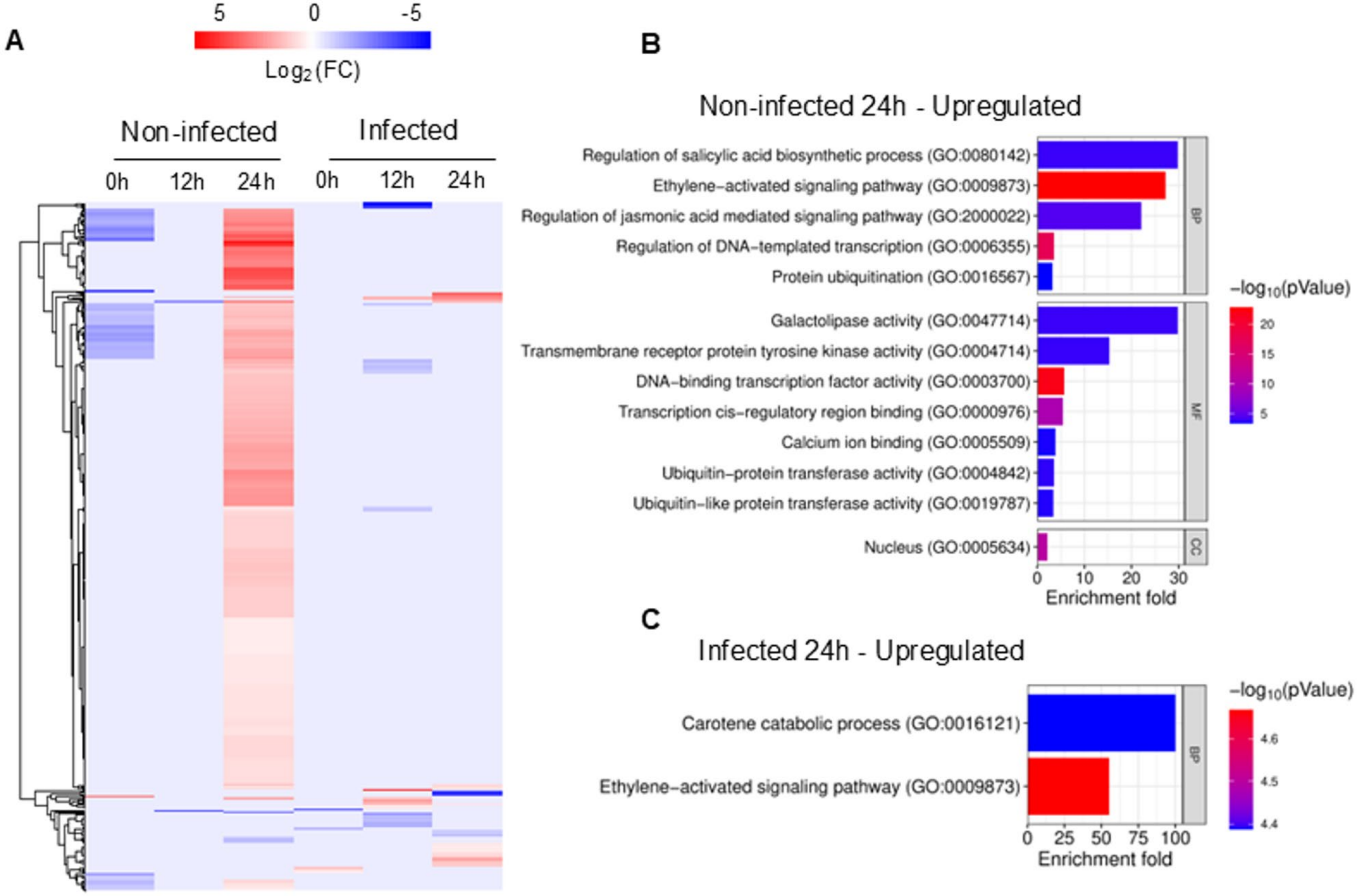
Transcriptomic responses triggered by SiO_2_ NP treatment and *P. viticola* infection. **A** Heatmap showing the expression profiles of all DEGs identified across all conditions and time points. Gene expression values are represented as log_2_ (fold change) relative to water-treated controls. Each row represents a gene, and each column represents a time point of the non-infected and infected conditions. Red and blue indicate upregulation and downregulation, respectively. **B-C** Gene ontology (GO) enrichment analysis of genes significantly upregulated in non-infected leaves at 24 hpt with SiO_2_ NPs (**B**) and of genes significantly upregulated in infected leaves at 24 hpi with *P. viticola* (**C**). The most enriched GO terms in the different categories are shown, with enrichment fold and -log_10_ (*p*-value) indicated by colour intensity

To further characterise grapevine responses to SiO_2_ NPs, the annotation of all DEGs was examined. A heatmap of the 505 DEGs was generated to visualise global expression patterns across treatments and time points (Fig. 6A). This representation provides an overview of the magnitude and distribution of transcriptional changes induced by SiO_2_ NP treatment, with samples separating according to infection status and time after application.

In non-infected samples at 0 hpt, none of the 82 downregulated genes could be clustered in any of the three gene ontology (GO) categories: biological process (BP), molecular function (MF) and cellular component (CC). Genes were then further investigated using the PANTHER gene list identification system, and 61 gene IDs could be identified. Among them, eight belonged to the family of AP2/ERF (ethylene-responsive) transcription factors (Vitvi03g00500, Vitvi04g00190, Vitvi11g00045, Vitvi16g01437, Vitvi16g01430, Vitvi16g01429, Vitvi19g01784, and Vitvi16g00380), four genes were identified as C2H2-type domain-containing protein (Vitvi06g01682, Vitvi03g00630, Vitvi18g00675, and Vitvi13g00262), and two were associated with ubiquitination processes, encoding for RING-type domain-containing protein (Vitvi05g00041) and RING-type E3 ubiquitin transferase (Vitvi06g00502) (Supplementary Table 1).

The strongest transcriptional activation occurred in non-infected leaves at 24 hpt, with 431 genes upregulated, of which 333 genes were unique to this condition (Supplementary Table 1). GO analysis indicated enrichment across BP, MF and CC categories (Fig. 6B). Enriched BP terms included “regulation of salicylic acid biosynthetic process” (GO:0080142; 3 genes), “ethylene-activated signalling pathway” (GO:0009873; 20 genes) and “regulation of jasmonic acid mediated signalling pathway” (GO:2000022; 4 genes), with fold enrichments of 29.77, 26.82 and 22.05, respectively (Fig. 6B).

Within the SA-associated category, *PAD4* (Vitvi07g01908) and *SARD1* (Vitvi17g00291, Vitvi17g00293), key regulators of SA biosynthesis and systemic acquired resistance (SAR), were induced [33, 34]. A large group of twenty genes related to the ET-activated signalling pathway corresponded to AP2/ERF transcription factors, of which many are involved in stress response regulation [35]. Among these, *ERF14* (Vitvi16g01429, Vitvi16g01430, Vitvi16g01432, Vitvi16g01434, Vitvi16g01437, Vitvi16g00362, Vitvi16g00363, Vitvi16g00370), *ERF1* (Vitvi16g00349, Vitvi15g01203) and other *ERFs* including *ERF109* (Vitvi03g00500, Vitvi07g02069), *ERF91* (Vitvi10g00522), *ERF4* (Vitvi19g011784) and *ERF5* (Vitvi16g00380) were identified (Supplementary Table 1). Genes encoding ACS6 (Vitvi02g00032), a key enzyme in ET biosynthesis and MYB44 (Vitvi03g00559), an ET-responsive transcription factor, were also upregulated, indicating the activation of biosynthetic and regulatory components of the ET pathway. Although fewer JA-related genes were detected, transcripts annotated as TIFY10B/JAZ2 (Vitvi11g00050) and MYC2 (Vitvi02g00698) were upregulated, suggesting a minor activation of the JA pathway.

Two additional BP terms were identified: “regulation of DNA-templated transcription” (GO:0006355; 58 genes) and “protein ubiquitination” (GO:0016567; 12 genes). Although these terms showed lower fold enrichment (3.62 and 3.19, respectively), the transcriptional regulation category included a substantial number of transcription factors associated with stress responses, including members of the WRKY, ERF, NAC, MYC and MYB families. WRKYs are key transcriptional regulators of plant immunity, acting downstream of SA and JA signalling to promote defence genes expression [36]. Among these, *WRKY33* (Vitvi06g00741, Vitvi08g00793), *WRKY6* (Vitvi10g00063), *WRKY30* (Vitvi16g01213), *WRKY40* (Vitvi09g01122), *WRKY51* (Vitvi04g01985), *WRKY53* (Vitvi15g01003) and *WRKY54* (Vitvi13g01916) were identified (Supplementary Table 1). Also, two genes coding for key regulators of stilbene biosynthesis, *MYB14* (Vitvi07g00598) and *MYB15* (Vitvi05g011733), were upregulated. Within the ubiquitination process category, five of the 12 genes encoded RING H2-type E3 ligases, including *ATL3* (Vitvi09g00012) and *ATL6* (Vitvi14g00275) (Supplementary Table 1).

In the MF category, the highest fold enrichments were observed for “galactolipase activity” (GO:0047714; 3 genes; 29.77-fold) and “transmembrane receptor protein tyrosine kinase activity” (GO:0004714; 4 genes; 15.27-fold). The term “calcium ion binding” (GO:0005509; 10 genes; 3.86-fold) included several calmodulin-like genes and one gene encoding a calcium-dependent protein kinase. Other MF and CC terms largely overlapped with BP results, such as DNA-binding, transcription factor activity, ubiquitin protein transferase activity, and nucleus (Fig. 6B).

In infected samples, the transcriptional response was much lower. There were few genes induced at 12 hpi, representing different functional categories, but were not particularly informative regarding resistance and stress-related functions (Supplementary Table 1). At 24 hpi, 29 genes were upregulated, and 8 genes downregulated (Fig. 5B). GO and enrichment analyses revealed two BP terms for the upregulated genes, “carotene catabolic process” (GO:0016121), with two genes, *NCED3* (Vitvi19g01356) and *NCED5* (Vitvi10g00821), as well as “ethylene-activated signalling pathway” (GO:0009873), with two genes encoding ERF transcription factors, *ERF109* (Vitvi07g02069) and *ERF53* (Vitvi12g00348) (Fig. 6C). A more precise identification of the remaining genes was then performed using the PANTHER platform. From this analysis, genes linked with diverse biological processes were identified and included genes coding for a NINJA-family protein (Vitvi07g01120), CYP94C1 (Vitvi06g01454), a tryptophan synthase (Vitvi10g00034), a terpene synthase-related protein (Vitvi12g00574), a hexokinase (Vitvi10g00037), a IME2-dependent signalling protein (Vitvi14g00063) and a heat stress transcription factor (Vitvi11g00339), among others (Supplementary Table 1).

### Accumulation of metabolites in response to SiO_2_ NPs and *P. viticola* infection

**Fig. 7:**
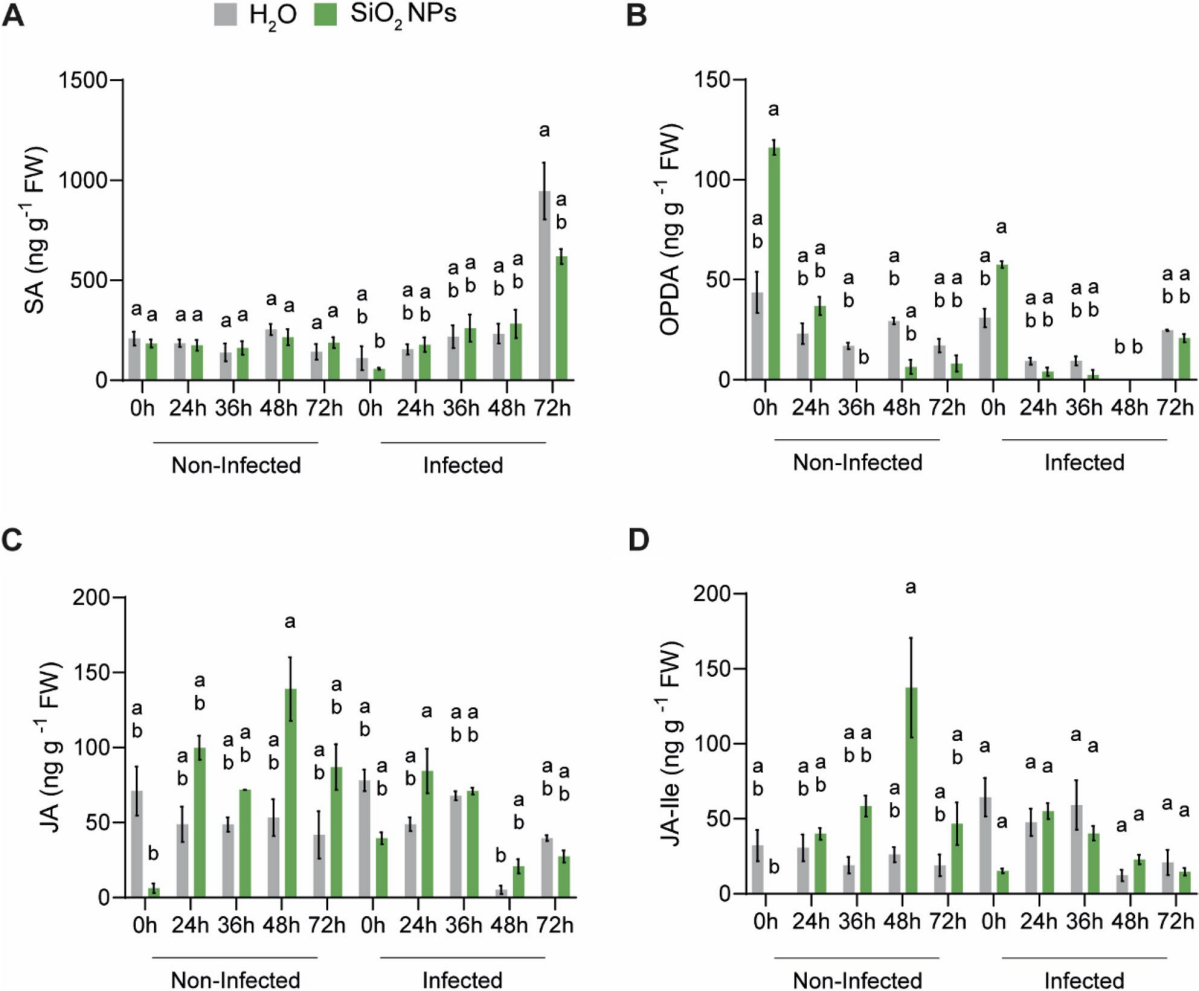
Quantification of phytohormones accumulation following SiO_2_ NP treatment and *P. viticola* infection. Levels of salicylic acid (SA) (**A**), 12-oxo-phytodioneic acid (OPDA) (**B**), jasmonic acid (JA) (**C**) and jasmonoyl-isoleucine (JA-Ile) (**D**) were measured by ultra-high performance liquid chromatography (UHPLC) in grapevine leaves treated with either water (H_2_O) or SiO_2_ NPs. Samples were collected at 0, 24, 36, 48 and 72 hpt and hpi for non-infected and infected conditions, respectively. Data represent means ± SE of three independent experiments (*n* = 3), each consisting of a pool of 30–35 leaf discs from four different leaves. Different letters indicate significant differences at *P* < 0.05 (Kruskal-Wallis followed by Dunn’s post hoc test with Holm adjustment for multiple comparisons) between treatments and time points within the same condition (non-infected or infected)

To gain insight into the metabolic changes in *V. vinifera* in response to SiO_2_ NP treatment, we quantified selected phytohormones and stilbenes in treated plants, with and without subsequent infection by *P. viticola*, at different time points (Fig. 5A). In response to SiO_2_ NP treatment alone (non-infected), levels of SA did not rise over time (Fig. 7A). However, when plants were infected with *P. viticola*, an increase in SA levels was observed at 72 hpi, with no significant differences between control and SiO_2_ NP-treated samples (Fig. 7A). Levels of the JA intermediate metabolite 12-oxo-phytodienoic acid (OPDA) showed a decrease over time from 0 h to 72 h in both non-infected and infected samples (Fig. 7B). On the contrary, levels of JA and JA-Ile tended to increase over time, reaching a peak at 48 hpt in non-infected samples (Fig. 7C-D). However, this effect was less pronounced in infected samples.

For all the stilbenes quantified, no accumulation was observed in non-infected samples, indicating that SiO_2_ NPs do not trigger the induction of these metabolites in our conditions (Supplementary Fig. 2). Levels of *cis*-ε-viniferin were not detected in any of the samples and time points (Supplementary Fig. 2D). However, in infected samples, a gradual accumulation of *trans*-piceid, *trans*-resveratrol, *trans*-pterostilbene, *trans*-ε-viniferin and *trans*-δ-viniferin was observed over time, with the highest levels detected at 72 hpi. Although not statistically significant, levels of stilbenes tended to be lower in SiO_2_ NP-treated samples compared to the water-treated controls (Supplementary Fig. 2).

## Discussion

### SiO_2_ NPs reduce *P. viticola* infection without direct toxicity

Our results in leaf disc assays demonstrate that treatment with low doses (0.2 and 0.5 g L^− 1^) of SiO_2_ NPs significantly reduced *P. viticola* infection, whereas the highest tested dose (1 g L^− 1^) was less effective (Fig. 1). Similar dose-dependent effects have been reported in *A. thaliana* and rice, where concentrations above 1 g L^− 1^ reduced resistance to bacterial and fungal pathogens, respectively [28, 30]. While we did not observe any visible phytotoxicity at the highest dose, the decreased efficacy may reflect a hormetic response. Hormesis is thought to involve fine-tuned regulation of defence signalling: at low doses, these pathways are optimally induced, enhancing resistance, whereas high doses may overstimulate signalling, triggering negative feedback that reduces defence effectiveness [29, 37, 38]. Excessive SiO_2_ NP exposure may lead to increased release of soluble Si(OH)_4_, potentially generating mild oxidative stress altering signalling pathways, as previously reported [28]. In our conditions, the reduced efficacy at 1 g L^− 1^ may therefore reflect a mild attenuation of defence signalling under higher nanoparticle exposure. This interpretation underscores the importance of optimising SiO_2_ NP concentration to maximise protective effects without overstimulation.

Importantly, SiO_2_ NPs reduced *P. viticola* infection without exhibiting direct toxicity, as both pathogen viability and infectivity remained unaffected, which is in line with previous studies showing no detrimental effects of SiO_2_ NPs on *P. syringae* and *M. oryzae* viability [28, 30]. However, contrasting evidence exists, as other studies have reported direct antifungal activity of SiO_2_ NPs against *Rhizoctonia solani* and *Alternaria solani*, with reduced mycelial growth observed following nanoparticle incubation [39, 40]. These discrepancies suggest that the antimicrobial effects of SiO_2_ NPs may be species-specific and dependent on pathogen physiology or environmental context.

### Field performance of SiO_2_ NPs under contrasting epidemic conditions

Across three consecutive years, SiO_2_ NP treatments reduced downy mildew incidence and severity on both foliage and bunches under low to moderate disease pressure (2023 and 2025), whereas efficacy declined under high epidemic pressure (2024). The 2024 season was characterised by climatic conditions highly favourable to downy mildew development. Under such intense and continuous infection pressure, repeated SiO_2_ NP applications were insufficient to fully contain *P. viticola* spread, indicating that their protective capacity may be exceeded when inoculum pressure and infection frequency are high. Notably, the organic reference also showed high disease incidence, although it reduced final severity to acceptable levels, suggesting that 2024 represented a borderline scenario for biological control strategies. These observations indicate that SiO_2_ NPs may be most suitable under low to moderate epidemic pressure or within integrated protection schemes. In situations where disease risk prediction models forecast sustained high infection pressure, combining SiO_2_ NPs with reduced or full doses of copper-based products may represent a realistic strategy to ensure grapevine protection while significantly decreasing overall fungicide input and environmental impact.

Results from our field trials are in line with those of Rashad et al. [31], who reported a significant reduction of downy mildew in *V. vinifera* cv. Thompson Seedless following foliar treatments with SiO_2_ NPs. Despite substantial differences in spray volume, nanoparticle deposition and environmental conditions compared with our trials (four applications delivering 10–20-fold higher nanoparticle amounts under arid Egyptian conditions), Rashad et al. reported approximately 75% efficacy on foliage. This reinforces that SiO_2_ NPs can reduce downy mildew severity across contrasting environments and application regimes, supporting their potential as an alternative source of protection in grapevine.

Beyond differences in spray volume and deposition, the timing of SiO_2_ NP application relative to infection events can play a critical role in determining field efficacy. It was previously reported that repeated pre-infection treatments enhance resistance by enabling faster defence gene activation upon pathogen attack [41]. In our trials, between two and five applications occurred before the appearance of the first symptoms, suggesting that the protection conferred by SiO_2_ NPs may partly rely on a priming effect. However, this hypothesis was not directly tested under field conditions and therefore remains speculative. Further studies are required to determine whether such a priming effect occurs under vineyard conditions and, if so, to clarify its duration and the optimal treatment interval for maintaining protection.

Field conditions at the time of application likely affected both nanoparticle uptake and overall efficacy. A fraction of SiO_2_ NPs penetrates grapevine leaves through the stomata and reaches the substomatal chamber within 24 h after application, as confirmed by our TEM observations. While these results demonstrate the internalisation of nanoparticles within leaf tissues, the relative proportion of internalised versus surface-retained particles could not be quantified, as accurate assessment of Si speciation and localisation in planta remains technically challenging [42, 43]. This aspect therefore requires further dedicated investigation. Owing to this internalisation mechanism, a short rain-free period following treatment may be sufficient for effective uptake, representing an advantage over conventional contact fungicides that require longer drying times.

### SiO_2_ NPs trigger the activation of defence-related genes

Regarding gene expression changes during treatment, the transcriptional response was the strongest in non-infected samples, with 88% of the DEGs (445 out of 505) detected across the three time points. At 0 hpt, 82 genes were downregulated, and only a single gene was upregulated. The biological relevance of this immediate repression is unclear. No gene ontology enrichment was detected for this gene set, indicating that the downregulated transcripts do not cluster into any coherent biological process, molecular function or cellular component. To our knowledge, such an early and broad transcript downregulation following nanoparticles or other elicitor treatments has not been previously reported but could correspond to a very rapid response to SiO_2_ NPs. Interestingly, many of these initially repressed genes were subsequently induced at 24 hpt, including four genes encoding C2H2-type zinc fingers transcription factors associated with methyl jasmonate, abscisic acid (ABA) and SA signalling in *V. vinifera* [44]. At 12 hpt, only two genes were downregulated, and none were upregulated, indicating that no clear transcriptional response to SiO_2_ NP treatment was detected at this intermediate time point under our conditions. A marked activation became evident only at 24 hpt, with 431 genes upregulated.

At 24 hpt, several upregulated genes were associated with the SA signalling pathway, such as *PAD4* and *SARD1*, previously associated with systemic acquired resistance (SAR) establishment [45]. While these results are in line with previous studies showing that SiO_2_ NPs induce SA accumulation and activate SA-related defences and SAR in other plant species [28, 46, 47], our metabolite analysis did not reveal a consistent increase in SA following foliar SiO_2_ NP treatment, which was rather associated with *P. viticola* infection. Discrepancies between our findings and previous reports may result from differences in experimental design, including the mode of application (foliar spray, irrigation, drop inoculation), the physico-chemical properties of the nanoparticles (type, size and concentration), or plant species-specific responses. Whether SiO_2_ NPs can elicit systemic SA-dependent responses in grapevine remains unclear.

Beyond SA signalling, genes related to ET and JA pathways were also induced. Notably, several ERF transcription factors with known defence functions were upregulated following SiO_2_ NP treatment. Among them, *ERF14*, which has not been previously characterised in grapevine, has been shown to activate pathogenesis-related genes, thereby contributing to antifungal defence in apple [48]. In *(A) thaliana*, *ERF14* overexpression enhances resistance to *Fusarium oxysporum* and induces the expression of *ERF1* [49]. Consistent with this regulatory link, two *ERF1* genes were also induced in our dataset. *ERF1* is a well-known regulator of defence and its overexpression in grape berries reduces susceptibility to *Botrytis cinerea* [50]. Several other *ERFs* induced by SiO_2_ NPs in our study have also been linked to stress or defence regulation in different species. These include *ERF109*, activated by *(B) cinerea* in grapevine [51], *ERF91* and *ERF4*, which regulate alkaloid and phenylpropanoid accumulation in tobacco, respectively [52, 53] and *ERF5*, implicated in early defence against *P. viticola* and *Erysiphe necator* (powdery mildew) in grapevine [54, 55]. Additional *ERFs* were induced as well, although their functions remain uncharacterised, highlighting potential new candidates for future functional analyses. The concurrent induction of *ACS6* and *MYB44*, two components associated with ET biosynthesis and signalling, further supports the activation of the ET pathway by SiO_2_ NPs. Interestingly, ET-related genes, including *ERFs*, are also activated during *P. viticola* infection in the resistant species *V. riparia* [56]. This suggests that a subset of the ET-associated regulators triggered by SiO_2_ NPs may be functionally relevant to the enhanced resistance observed in SiO_2_ NP-treated grapevine.

Only few JA-related genes were induced by SiO_2_ NPs, including *TIFY10B/JAZ2* and the master regulator *MYC2*, indicating that components of the JA pathway were engaged [57, 58]. Jasmonates profiling showed no significant changes, although SiO_2_ NP-treated leaves tended to accumulate slightly more JA and JA-Ile, while OPDA levels decreased over time, consistent with its conversion into downstream jasmonates. The induction of a 12-oxo-phytodienoate reductase (Vitvi18g03161) further supports the activation of JA biosynthesis observed in this study. This is in line with a recent report showing an accumulation of JA levels in rice leaves following SiO_2_ NP application [59]. Although these effects were modest and likely influenced by the limited number of replicates, they suggest that SiO_2_ NPs elicit a weak JA-associated response that may contribute to the enhanced resistance observed, consistent with previous studies implicating JA signalling in grapevine defence against *P. viticola* [56, 60–62].

Beyond hormone-related regulators, several WRKY transcription factors were also induced by SiO_2_ NPs. Among them, *WRKY33*, known to contribute to resistance against *P. viticola* in grapevine [63], as well as *WRKY6*, *WRKY30* and *WRKY51*, which modulate SA, ET and ROS pathways [64, 65], point to the engagement of defence-associated WRKY modules. The induction of *WRKY54*, a positive regulator of *SARD1* in *A. thaliana* [66], and *WRKY40*, a known negative regulator of immunity [67], indicates that both activating and repressive WRKY branches are transiently activated by SiO_2_ NPs. The induction of *WRKYs* is consistent with the activation of upstream MAPK signalling [68]. *MPK3* was also upregulated, and in *A. thaliana* MPK3 phosphorylates WRKY33 to trigger antifungal defence and ET production via ACS6 [69, 70]. The simultaneous induction of *MPK3*, *WRKY33* and *ACS6* in our dataset therefore suggests that SiO_2_ NPs activate a conserved MAPK-WRKY-ET signalling module in grapevine.

In addition, several calcium sensor genes (CMLs and one CDPK) were induced by SiO_2_ NPs, indicating activation of calcium-dependent signalling. Because *CMLs*, *CDPKs*, *MYB14* and *MYB15*, and *WRKY53* are all associated with stilbene biosynthesis [71–74], their coordinated induction suggests that SiO_2_ NPs induce the early steps of this pathway. However, stilbene levels did not increase in SiO_2_ NP-treated plants, contrasting with the strong accumulation observed after *P. viticola* infection, indicating that metabolite production likely requires pathogen-derived cues. Finally, the induction of two RING-H2 E3 ubiquitin ligases (*ATL3* and *ATL6*) suggests that post-translational regulation may also contribute to SiO_2_ NP-induced responses.

Taken together, these results indicate that SiO_2_ NPs activate a broad defence-associated response in grapevine, involving early signalling components (MAPK and calcium-dependent signalling), hormone pathways (SA, JA, ET) and multiple families of defence-related transcription factors. This pattern resembles the early transcriptional reprogramming typically associated with elicitor perception, suggesting that although classical plant defence metabolites were not significantly induced up to 72 hpt, SiO_2_ NPs can transiently activate core elements of the grapevine immune network.

### SiO_2_ NP-induced defence genes are not activated upon *P. viticola* infection

In SiO_2_ NP-treated plants sampled 24 h after *P. viticola* inoculation, most hormone- and defence-associated genes were not induced. The few upregulated genes were linked to diverse biological processes and might reflect general physiological responses rather than defence activation. The induction of a gene annotated as a *NINJA*-family protein, known to be a repressor of JA signalling [75], together with that of *CYP94C1*, which is involved in JA-Ile catabolism in *A. thaliana* [76], may indicate a possible attenuation of JA-mediated defences. In addition, the upregulation of *NCED3* and *NCED5*, coding for 9-cis-epoxycarotenoid dioxygenases involved in ABA biosynthesis, may indicate a shift towards ABA accumulation, which is known to antagonise JA/ET-dependent pathways [77].

The strong attenuation of transcriptional activation during infection indicates that defence-related genes upregulated in non-infected leaves are not maintained upon *P. viticola* infection. This suggests that *P. viticola* infection overrides or suppresses the transcriptional response initially triggered by SiO_2_ NPs. Indeed, *P. viticola* is known to deliver effectors that interfere with host immunity [1, 78], which may dampen or mask parts of the SiO_2_ NP-triggered response. However, despite this attenuation, SiO_2_ NP-mediated protection was still observed in both leaf discs assays and field trials. Therefore, the functional significance of the transcriptional changes observed in non-infected tissues for disease resistance remains to be determined, implying that additional mechanisms must contribute to this response.

One possibility is that SiO_2_ NPs induce a very early and transient defence response, occurring within minutes to a few hours after treatment or infection, which may not have been captured by our first sampling time at 12 h. In other plant systems, SiO_2_ NPs have been reported to stimulate ROS production, which can contribute to pathogen inhibition through oxidative damage to pathogen proteins and cellular structures [79, 80]. However, ROS accumulation was not quantified in grapevine at early time points in the present study, and its potential contribution therefore remains hypothetical. In addition, SiO_2_ NP treatment may lead to the accumulation of defence-related metabolites that were not included in our targeted analysis. Previous metabolomic analyses in grapevine have demonstrated the ability to identify and quantify a wide range of secondary metabolites associated with defence [81]. Untargeted metabolomic approaches in SiO_2_ NP-treated and infected leaves would therefore be useful to identify potential bioactive compounds that could contribute to the observed protection. Future experiments including early ROS measurements, transcriptomic analyses at earlier time points, and extensive metabolomic profiling will be necessary to better characterise grapevine responses to SiO_2_ NPs.

**Fig. 8:**
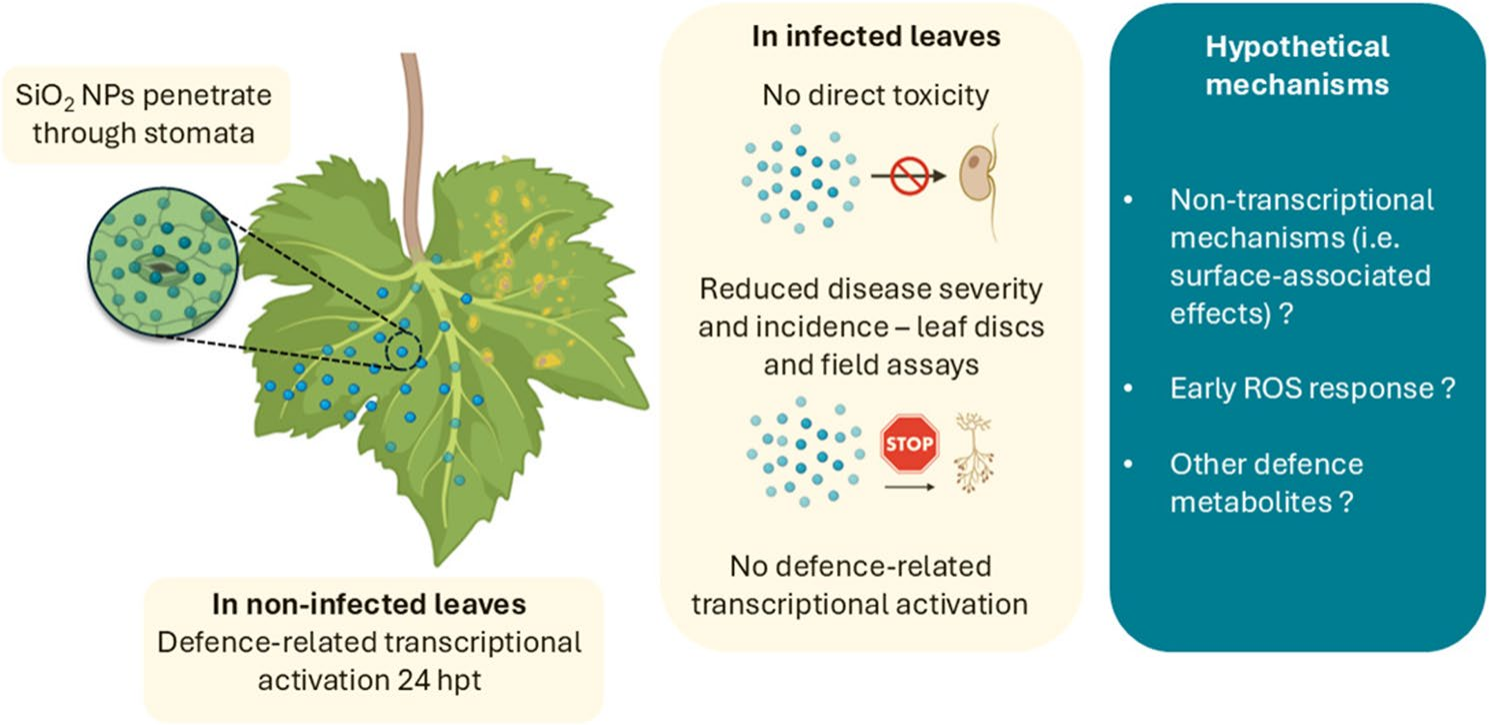
Schematic model summarising the demonstrated and proposed mechanisms underlying SiO_2_ NP-mediated reduction of *P. viticola* infection in grapevine

Alternatively, SiO_2_ NPs may confer protection through non-transcriptional mechanisms, including rapid post-translational regulation of signalling proteins [82] or changes in leaf surface properties, stomatal behaviour, or callose deposition, all of which can impede pathogen entry [83]. However, these processes were not directly assessed in the present study, and their contribution therefore remains uncertain, as no protein-level or phosphorylation analyses were performed to support a role for post-translational regulation. Finally, although TEM images indicate the accumulation of SiO_2_ NPs at stomatal openings and on the outer cuticular surface, they do not provide clear evidence of stomatal clogging or physical blockage. This suggests that any interference with pathogen entry is likely partial or indirect rather than purely mechanical. Attempts to visualise direct interactions between SiO_2_ NPs and *P. viticola* at the stomatal interface were inconclusive. Further high-resolution imaging and functional analyses will therefore be required to determine whether such non-transcriptional and surface-associated effects contribute to limiting pathogen penetration. The experimentally demonstrated and hypothesised mechanisms contributing to SiO_2_ NP-mediated protection are integrated in a schematic model (Fig. 8).

## Conclusion

This study provides new insights into how SiO_2_ NPs reduce downy mildew symptoms in *V. vinifera* and into the associated molecular responses of grapevine. Across controlled experiments and field trials, SiO_2_ NPs consistently decreased infection by *P. viticola* without exhibiting direct toxicity to the pathogen. Transcriptomic analyses revealed transient transcriptional reprogramming in non-infected leaves, activating signalling components, hormone-related regulators, and defence-associated transcription factors. However, these responses were not maintained during pathogen challenge, and classical defence markers alone do not fully explain the observed protection, suggesting that additional mechanisms, currently unidentified, may contribute (Fig. 8).

By combining microscopy, gene expression, metabolite profiling, and multi-year field evaluations, this work highlights the potential of SiO_2_ NPs as a promising tool for sustainable downy mildew management under moderate disease pressure and diverse environmental conditions. Further studies are needed to clarify their molecular modes of action and to optimise application strategies. In the context of reduced copper use, combining SiO_2_ NPs with lower copper doses could provide complementary modes of action and achieve effective disease control while minimising environmental impact and production costs.

## Methods

### Plant growth conditions

All experiments were conducted with cuttings of *V. vinifera* cv. Cabernet Sauvignon. The vines used in this work were propagated through hardwood cuttings. The wood was obtained from certified vineyards of the Agroscope clonal selection program. Cuttings were grown in pots of 1 L (Ø 13 cm) containing a peat-rich substrate mix (55% blonde peat, 10% compost, 10% coconut fiber, 15% topsoil and 10% perlite). After 14–16 weeks of vegetative growth under standard greenhouse conditions (20 °C day/18°C night, 50–60% humidity with supplemental lighting from September to May: 100 W m^− 2^ for 12 h daily), plants were used for experiments.

### SiO_2_ NP synthesis and visualisation within the leaf

SiO_2_ NPs, synthesised and characterised as described by El-Shetehy et al. [28], were kindly provided by Dr Fabienne Schwab. Briefly, one equivalent of tetraethyl orthosilicate (Merck, Germany) was added to an equilibrated reaction mixture at 70 °C containing two equivalents of ultrapure water and absolute ethanol. After 3 h of hydrolysis and polycondensation of tetraethyl orthosilicate, the particles obtained were washed three times by centrifugation (15,000 *g* for 15 min) in ultrapure water followed by five or more steps of dialysis through a membrane of 14 kDa (Carl Roth, Germany). Batches of nanoparticles with hydrodynamic diameter in the range of 64.8–76.7 nm were prepared. Dynamic light scattering was used to quantify the hydrodynamic size and surface charge of the diluted samples at 1% v/v (NanoBrook Particle Size Analyzer 90Plus, Brookhaven Instruments, USA). Inductively coupled plasma-optical emission spectroscopy and gravimetry were used to quantify the SiO_2_ concentration [42].

To visualise the SiO_2_ NPs in grapevine leaves, transmission electron microscopy was used. Detached leaves were sprayed with water (control) and SiO_2_ NPs at 5 g L^− 1^ and samples of approximately 1–2 mm^2^ were cut 24 h later. The samples were then pre-fixed in a solution of 3% glutaraldehyde and 2% paraformaldehyde in 0.14 M PIPES buffer (pH 7.0) under vacuum infiltration at room temperature. Samples were rinsed three times for 10 min each in 0.07 M PIPES buffer, embedded in 2% agarose, and post-fixed for 1.5 h in 1% OsO_4_ in 0.07 M PIPES buffer at room temperature. After three washes in distilled water, samples were dehydrated using an ethanol series (30%, 50%, 70%, 95% and 100%), infiltrated in a solution of ethanol mixed with propylene oxide (1:1) and pure propylene oxide, followed by a series of epoxy resin (DER736, EMbed 812, NMA and BDMA 2.5%) via graded resin-propylene oxide mixtures (1:3 for 2 h, 1:1 for 2 h and 3:1 for 17 h). Final infiltration was performed in 100% epoxy resin for 6 h, followed by polymerisation at 60 °C for 48 h. Semi-thin Sect. (0.5–1 μm) were stained by floating on a solution of 1% methylene blue, 1% sodium borate and 1% azure II at 50 °C for 10 s. Ultrathin Sect. (80 nm) were prepared with an ultramicrotome (Leica EM UC7) and subsequently stained with 2% uranyl acetate for 15 min followed by lead citrate for 5 min. The TEM images of ultrathin sections were taken on a Tecnai G2 Spirit BioTwin transmission electron microscope (Thermo Fisher Scientific, USA) fitted with an Eagle V CCD camera (Raptor Photonics, United Kingdom).

### *Plasmopara viticola* culture and infection assays

*P. viticola* sporangia were collected from sporulating lesions of artificially infected leaves of *V. vinifera* cv. Cabernet Sauvignon by vacuum aspiration using a filtered tip. A sporangia solution was generated and, using a Thoma cell (haemocytometer), adjusted to 2 × 10^5^ sporangia mL^− 1^ in a 50 mL Falcon tube containing ultrapure water and gently stirred for 2 h at room temperature in darkness. As soon as the zoospores were released, the suspension was used to inoculate leaf tissues.

For inoculation, leaf discs (Ø 18 mm) were punched out of leaves with a cork borer and placed on a wet filter paper in Petri dishes (Ø 90 mm). Petri dishes containing 10 leaf discs were then treated on their abaxial side with 5 mL of SiO_2_ NPs at different concentrations (0.2, 0.5 and 1 g L^− 1^) using a Potter precision spray tower (Burkard Manufacturing Co Ltd, United Kingdom). Water and a 0.0625% copper hydroxide (Cu(OH)_2_) solution (Kocide^®^ Opti, Bayer, Switzerland) were used as negative and positive controls, respectively. Twenty-four hours later, leaf discs were inoculated with a *P. viticola* zoospores solution (5 × 10^4^ zoospores mL^− 1^) using a custom-made glass spray and the percentage of sporulating area was quantified under a light microscope at 7 dpi. For each treatment, 20 leaf discs were used per independent experiment, distributed in 2 Petri dishes with 10 discs each. Experiments were repeated independently three times, resulting in three biological replicates per treatment and a total of 60 leaf discs.

### Fungal toxicity assays

The direct effect of SiO_2_ NPs on *P. viticola* was analysed using two complementary approaches. First, zoospore motility was assessed by mixing equal volume of SiO_2_ NPs and sporangia suspension for 2 h at room temperature. Both solutions were prepared at twice their target concentrations, so that after mixing, the final concentrations were 2 × 10^4^ sporangia mL^− 1^ and 0.2 g L^− 1^ SiO_2_ NPs. Water and Cu(OH)_2_ (0.0625%) served as negative and positive controls, respectively. For each treatment, three independent mixtures were prepared per experiment. For each mixture, zoospore motility was assessed in three separate counts using a Thoma cell. Experiments were repeated independently three times, resulting in three biological replicates per treatment, with three technical observations per mixture.

The same mixtures were then used to inoculate leaf discs (Ø 18 mm) to evaluate whether zoospores could develop an infection after contact with the tested products. Four 20 µL drops of the mixture were deposited on the abaxial side of each disc. Seven days post-inoculation, leaf discs were collected and placed in a 15 mL Falcon tube filled with 2 mL water and vortexed. Released sporangia were counted using a Thoma cell, and results were expressed as number of sporangia per cm^2^ of leaf tissue. For each treatment, 20 leaf discs were used per independent experiment, distributed in 2 Petri dishes with 10 discs each. Experiments were repeated independently three times, resulting in three biological replicates per treatment and a total of 60 leaf discs.

### Experimental field trials

Three independent trials were conducted in Switzerland: in the experimental vineyards of Agroscope in 2023 (46°23’54’’N, 6°13’53’’E) and of FiBL in 2024 and 2025 (46°31’07’’N, 6°29’14’’E). The trials were arranged in a completely randomised block design on the susceptible cultivar *V. vinifera* cv. Chasselas. Phenological stages were determined according to the BBCH scale (an acronym derived from the German names of the coordinating institutions: Biologische Bundesanstalt, Bundessortenamt, and the chemical industry) [84].

Treatments were organised in four replicates (blocks), each consisting of 10 vines, and included an untreated control and an organic reference. Weather data were recorded throughout the season using a Campbell CR1000 weather station located near the plots. SiO_2_ NPs were applied at 0.2 g L^− 1^ throughout the season in combination with wettable sulfur (Thiovit^®^ Jet, Syngenta, Switzerland) at 0.4% to ensure powdery mildew (*E. necator*) control, as the primary focus was the efficacy against downy mildew. The organic reference consisted of copper hydroxide (Kocide^®^ Opti) at 250 g ha^− 1^ combined with wettable sulfur at 0.4%. Applications were made 11, 15 and 9 times in 2023, 2024 and 2025, respectively, according to predicted disease risk (www.agrometeo.ch). Spray volume was adjusted to canopy size, ranging from 200 L ha^− 1^ at the beginning of the season to 450 L ha^− 1^ at full leaf development. Phytotoxicity was monitored throughout the trial on young and old leaves, inflorescences, and bunches.

Disease assessments on leaves and bunches were conducted three to four times per season by recording disease incidence (proportion of organs showing symptoms) and disease severity (proportion of diseased leaf or bunch area) for downy mildew. For each assessment date, 100 leaves and 50 bunches per block were randomly evaluated, resulting in a total of 400 leaves and 200 bunches per treatment. Disease severity was scored on each organ using a 0–5 scale corresponding to increasing levels of symptom development. The summed scores were used to calculate incidence and severity indices, expressed as percentages. Percentage data were then subjected to an arcsine square-root transformation prior to analysis of variance to meet the assumptions of the test. The area under the disease progress curve (AUDPC) was calculated using the trapezoidal method [32], integrating disease incidence and severity over time to quantify cumulative disease development. Treatment efficacy (%) was calculated as: Efficacy = ((C-T)/C) x 100, where C is the mean AUDPC of untreated vines and T is the mean AUDPC of treated vines.

### Plant treatment and infection procedures for RNA-seq and defence metabolite quantification

Cuttings of identical age (4 months old) and comparable growth (18–20 leaves) were used. Detached leaves of similar size (5-6th leaves from the apex) were placed on moist filter paper in reusable plastic plates. For each condition and time point, four leaves (from four different plants) were treated on the abaxial side with fifty 20 µL drops of either water or SiO_2_ NPs at 0.2 g L^− 1^.

To assess the effects of SiO_2_ NP treatment alone (non-infected samples), leaf discs (Ø 4 mm) were harvested from treated areas at 0, 12 and 24 hpt for RNA extraction, and at 0, 24, 36, 48 and 72 hpt for defence metabolite quantification.

For infection assays (infected samples), leaves were treated as above. After 24 h, drops were removed with a sterile cotton bud and replaced by a *P. viticola* sporangia suspension (8 × 10^4^ sporangia mL^− 1^). Leaf discs (Ø 4 mm) were harvested from the infection sites at 0, 12 and 24 hpi for RNA extraction, and at 0, 24, 36, 48 and 72 hpi for defence metabolite quantification.

All sampling was carried out simultaneously by a coordinated team to ensure synchronised and consistent collection of localised responses. Discs were pooled per condition and time point and immediately frozen in liquid nitrogen.

A schematic summary of the experimental design is shown in Fig. 5A.

### RNA-sequencing and data analysis

Pooled frozen leaf discs were ground using a TissueLyser II (Qiagen, Germany). Total RNA was extracted from 100 mg of powdered tissue using the Spectrum™ Plant Total RNA Kit (Merck, Germany), following manufacturer’s instructions. The RNA concentration, purity and integrity were measured with an Agilent 2100 Bioanalyzer system (Agilent Technologies, USA). The RNA samples, with three biological replicates for each condition, were sent to Macrogen (The Netherlands) for library construction and sequencing. cDNA libraries were constructed from the total RNA samples using the TruSeq Stranded mRNA Library Prep Kit and sequencing was performed using an Illumina NovaSeq X Plus platform. Reads with a paired-end length of 151 bp were generated. The quality of raw RNA-seq reads was assessed using FastQC v0.11.7 [85]. Low quality reads and adapter sequences were removed using Trimmomatic v0.38 [86] and cleaned reads were aligned to the *V. vinifera* reference genome PN40024 12X v2 using HISAT2 v2.1.0 [87]. Transcript assembly and quantification were performed with StringTie v2.1.3b [88] and expression levels were reported as read counts and normalised as Fragments Per Kilobase of transcript per Million mapped reads (FPKM) and Transcripts Per Million (TPM). Prior to the calculation of DEGs, a normalisation step correcting sequencing depth bias was performed through the DIANE platform using TCC R package and DESeq2 method [89]. Genes with count sum below the threshold of 360 (10 times the sample number) were filtered out. Differential gene expression was performed using DESeq2 with |log_2_ (FC)| > 1 and *P* < 0.05 as thresholds through the DIANE platform. PCA analysis was performed on regularised log transformed read counts using R platform.

A statistical overrepresentation test was performed on the PANTHER classification system [90]. Different lists of genes of interest were uploaded to the workspace and compared to the *V. vinifera* genome (version of June 2024) using the Fisher’s Exact test type, with a false discovery rate of *P* < 0.05 and an absolute fold enrichment above 2. The results were exposed in enrichment plots created with the SRplot platform [91].

### Phytohormones and stilbenes quantification

For both stilbene and phytohormone quantification, 30 mg of powdered frozen leaf tissue was extracted with 200 µL methanol containing internal standards for SA, OPDA, JA and JA-Ile (each at a final concentration of 10 ng mL^− 1^). Samples were incubated at 60 °C for 15 min under agitation (900 rpm), rapidly cooled on ice for 5 min, and centrifuged. The supernatant was collected and transferred to UHPLC vials.

Chromatographic separation was performed using a Vanquish UHPLC system (Thermo Fisher Scientific, USA) equipped with a Hypersil Gold Column (1.9 μm, 50 × 2.1 mm) and a 0.2 μm inline filter. The mobile phases consisted of water with 0.1% formic acid (A) and acetonitrile with 0.1% formic acid (B). The gradient was as follows: 5% B initially; increased to 23% B over 0.7 min; to 26% B from 0.7 to 9 min; to 80% B from 9 to 13 min; and to 99% B from 13 to 14 min. The flow rate was 0.3 mL min^− 1^, injection volume 2 µL, and column temperature 35 °C.

Stilbenes were quantified using a UV diode-array detector at 310 nm and identified based on retention time and UV spectra compared with authentic standards. Phytohormones were analysed under identical chromatographic conditions using a TSQ Quantis Plus mass spectrometer (Thermo Fisher Scientific, USA) operating in electrospray ionisation mode. Spray voltage was set to 4200 V (positive) and 3800 V (negative) with sheath gas at 60, auxiliary gas at 15, sweep gas at 2, and ion transfer tube and vaporiser temperatures at 350 °C. Calibration curves were generated using authentic standards, and quantification was performed using internal standard normalisation. Deuterated standards were used when available (SA-D_4_ for SA and JA-D_5_ for JA). For OPDA and JA-Ile, deuterated analogues were unavailable, and SA-D_4_ was used as a substitute internal standard.

Internal standards and reference compounds were obtained from the following sources: Cayman (USA): JA, JA-Ile, OPDA, *trans*-piceid; Supelco (USA): SA, SA-D_4_; Toronto Research Chemicals (Canada): JA-D_5_; APIChem (China): *trans*-resveratrol, *trans*-pterostilbene. Viniferin standards included *trans*-δ-viniferin synthesised in-house and *trans*-ε-viniferin isolated from grapevine canes [92].

### Statistical analyses

Data were analysed using GraphPad Prism v.10.0 and Rstudio (R v.4.4.1) software. Normal distribution and variance homogeneity of data were analysed with the Shapiro-Wilk test and Levene’s test, respectively. If not normal, data were analysed using non-parametric tests.

For *P. viticola* infection assays, data were analysed using a One-Way ANOVA to assess differences among treatments. When significant differences were detected (*P* < 0.05), Tukey’s Honest Significant Difference post hoc test was applied to determine pairwise comparisons between treatments.

For field experimental trials, data were analysed using One-Way ANOVA to assess differences among treatments. When significant differences were detected (*P* < 0.05), Tukey’s Honest Significant Difference post hoc test was applied to determine pairwise comparisons between treatments. Prior to analysis, data were arcsine square-root-transformed to meet normality assumptions.

For phytohormones and stilbenes quantification, data were analysed using a Kruskal-Wallis test to assess differences among treatments and time points within the same infection condition (non-infected and infected analysed separately). When significant differences were detected (*P* < 0.05), Dunn’s post hoc test was applied to determine pairwise comparisons among treatments/time points.

Data are presented as means ± SE of three independent experiments and statistical significance is indicated by different letters.

## Supplementary Information

Supplementary Material 1: Supplementary Table 1. List of genes differentially expressed across all conditions. Log2(FC) values are represented for all conditions and timepoints.

Supplementary Material 2: Supplementary Fig S1. Overview of transcriptomic variation and overlap of DEGs. (A) Principal component analysis (PCA) of RNA-seq data showing the global transcriptomic variation among samples across treatments (H_2_O or SiO_2_ NPs), infection status (non-infected (NI) and infected (I)) and time points (0, 12 and 24 hpt and hpi). (B) Venn diagrams displaying the number of DEGs (|log2(FC)| > 1, *P* < 0.05) at 0, 12 and 24 h in non-infected (top) and infected (bottom) conditions, following SiO_2_ NP treatment compared to the respective water controls. Supplementary Fig S2. Quantification of stilbenes accumulation following SiO_2_ NP treatment and *P. viticola* infection. Levels of *trans*-piceid (A), *trans*-resveratrol (B), *trans*-pterostilbene (C), *cis-ε*-viniferin (D), *trans-ε*-viniferin (E) and *trans*-δ-viniferin (F) were measured by ultra-high performance liquid chromatography (UHPLC) in grapevine leaves treated with either water (H_2_O) or SiO_2_ NPs. Samples were collected at 0, 24, 36, 48 and 72 hpt and hpi for non-infected and infected conditions, respectively. Data represent means ± SE of three independent experiments (n=3), each consisting of a pool of 30-35 leaf discs from four different leaves. Different letters indicate significant differences at *P* < 0.05 (Kruskal-Wallis followed by Dunn’s post hoc test with Holm adjustment for multiple comparisons) between treatments and time points within the same condition (non-infected or infected).

## Data Availability

Raw RNA-seq data have been deposited in NCBI’s Gene Expression Omnibus [93] and are accessible through GEO Series accession number GSE312954 (https://www.ncbi.nlm.nih.gov/geo/query/acc.cgi?acc=GSE312954).

## Abbreviations

AUDPC: Area under the disease progress curve
BBCH: Biologische Bundesanstalt, Bundessortenamt and Chemical industry (phenological growth stage scale)
BP: Biological process
CC: Cellular component
Cu(OH)_2_: Copper hydroxide
DEG: Differentially expressed gene
ERF: Ethylene responsive factor
ET: Ethylene
GO: Gene ontology
hpi: Hours post-infection
hpt: Hours post-treatment
I: Infected
JA: Jasmonic acid
MF: Molecular function
NI: Non-infected
PCA: Principal component analysis
ROS: Reactive oxygen species
S: Sulfur
SA: Salicylic acid
SE: Standard error
Si: Silicon
SiO_2_: Silica
Si(OH)_4_: Monosilicic acid
SiO_2_ NPs: Silica nanoparticles
TEM: Transmission electron microscopy
UHPLC: Ultra-high performance liquid chromatography
