# Supplementary material for: Silica nanoparticles enhance resistance of Vitis vinifera to downy mildew
Source: BMC Plant Biol. 2026 Apr 15;26:906. doi: 10.1186/s12870-026-08718-0 (PMC13195864; doi:10.1186/s12870-026-08718-0)
Supplement: Supplementary file 2 — Supplementary Material 2: Supplementary Fig S1. Overview of transcriptomic variation and overlap of DEGs. (A) Principal component analysis (PCA) of RNA-seq data showing the global transcriptomic variation among samples across treatments (H2O or SiO2 NPs), infection status (non-infected (NI) and infected (I)) and time points (0, 12 and 24 hpt and hpi). (B) Venn diagrams displaying the number of DEGs (|log2(FC)| > 1, P < 0.05) at 0, 12 and 24 h in non-infected (top) and infected (bottom) conditions, following SiO2 NP treatment compared to the respective water controls. Supplementary Fig S2. Quantification of stilbenes accumulation following SiO2 NP treatment and P. viticola infection. Levels of trans-piceid (A), trans-resveratrol (B), trans-pterostilbene (C), cis-ε-viniferin (D), trans-ε-viniferin (E) and trans-δ-viniferin (F) were measured by ultra-high performance liquid chromatography (UHPLC) in grapevine leaves treated with either water (H2O) or SiO2 NPs. Samples were collected at 0, 24, 36, 48 and 72 hpt and hpi for non-infected and infected conditions, respectively. Data represent means ± SE of three independent experiments (n=3), each consisting of a pool of 30-35 leaf discs from four different leaves. Different letters indicate significant differences at P < 0.05 (Kruskal-Wallis followed by Dunn’s post hoc test with Holm adjustment for multiple comparisons) between treatments and time points within the same condition (non-infected or infected). [file 12870_2026_8718_MOESM2_ESM.docx]

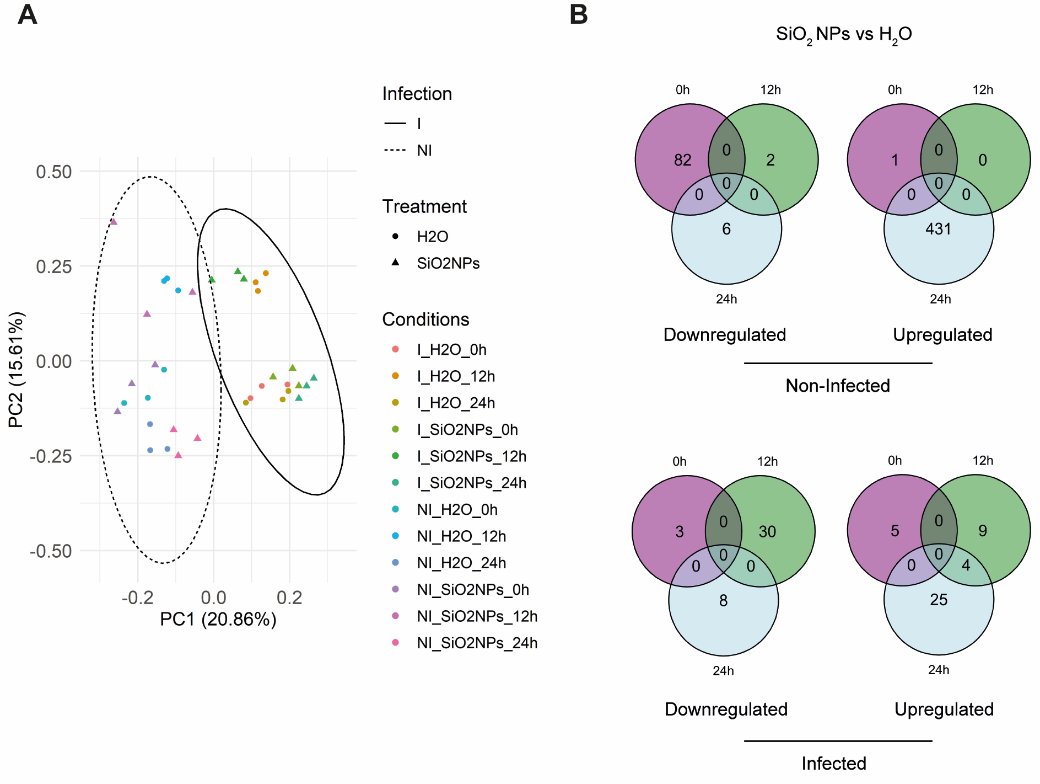


**Supplementary Figure 1.** Overview of transcriptomic variation and overlap of DEGs. **(A)** Principal component analysis (PCA) of RNA-seq data showing the global transcriptomic variation among samples across treatments (H_2_O or SiO_2_ NPs), infection status (non-infected (NI) and infected (I)) and time points (0, 12 and 24 hpt and hpi). **(B)** Venn diagrams displaying the number of DEGs (|log_2_(FC)| > 1, *P* < 0.05) at 0, 12 and 24 h in non-infected (top) and infected (bottom) conditions, following SiO_2_ NP treatment compared to the respective water controls.


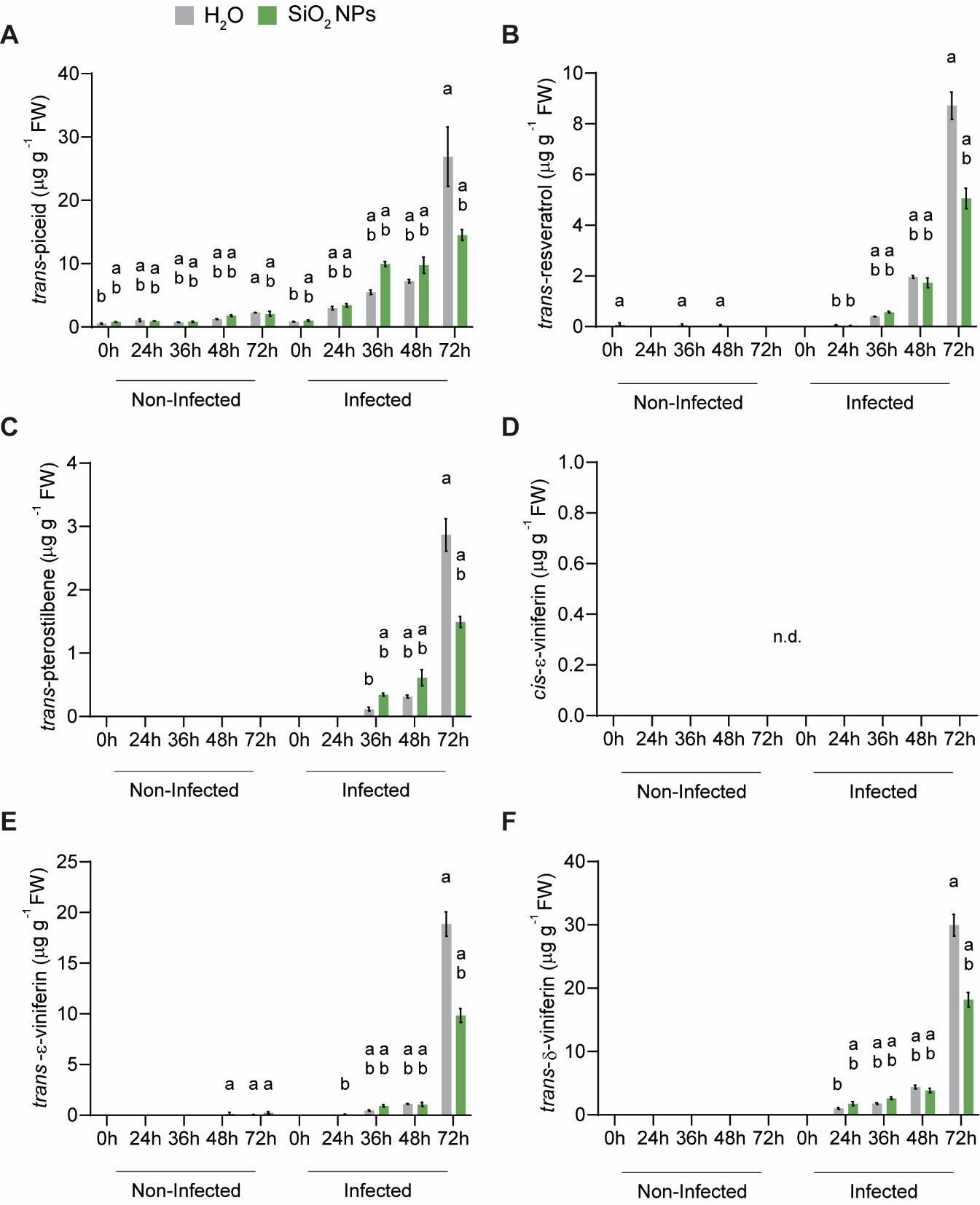


**Supplementary Figure 2.** Quantification of stilbenes accumulation following SiO_2_ NP treatment and *P. viticola* infection. Levels of *trans*-piceid **(A)**, *trans*-resveratrol **(B)**, *trans*-pterostilbene **(C)**, *cis*-ε-viniferin **(D)**, *trans*-ε-viniferin **(E)** and *trans*-δ-viniferin **(F)** were measured by ultra-high performance liquid chromatography (UHPLC) in grapevine leaves treated with either water (H_2_O) or SiO_2_ NPs. Samples were collected at 0, 24, 36, 48 and 72 hpt and hpi for non-infected and infected conditions, respectively. Data represent means ± SE of three independent experiments (n=3), each consisting of a pool of 30-35 leaf discs from four different leaves. Different letters indicate significant differences at *P* < 0.05 (Kruskal-Wallis followed by Dunn’s post hoc test with Holm adjustment for multiple comparisons) between treatments and time points within the same condition (non-infected or infected).
